# Apple Pomace Compositional Data Highlighting the Proportional Contribution of Polymeric Procyanidins

**DOI:** 10.3390/molecules28145494

**Published:** 2023-07-18

**Authors:** Keren Bindon, Song Qi, Stella Kassara, Luca Nicolotti, Alicia Jouin, Maggie Beer

**Affiliations:** 1 The Australian Wine Research Institute, Waite Precinct, Hartley Grove cnr Paratoo Road, Glen Osmond, Adelaide, SA 5064, Australia; 2 Metabolomics Australia (SA Node), Glen Osmond, Adelaide, SA 5064, Australia; 3 Institute of Vine and Wine Science, The University of Bordeaux, 210 Chemin de Leysotte, 33882 Villenave d’Ornon, France; 4 The Maggie Beer Foundation, SAHMRI, Adelaide, SA 5001, Australia

**Keywords:** *Malus domestica*, fibre, procyanidin, phloretin, quercetin, flavan-3-ol, chlorogenic acid, molecular mass, polymerisation, pectin, lignin, antioxidant

## Abstract

Recent years have seen an increase in research focusing on the amelioration of apple pomace waste for use in the food and nutraceutical industries. Much of this work has concentrated on the characterisation of the polyphenol composition of apple pomace materials to determine their role in conferring nutritional and health benefits. Although apples contain substantial quantities of polymeric procyanidins (condensed tannins), this class of compounds has received limited attention in apple research. This study quantified the polymeric procyanidins in apple pomace extracts using a rapid, methyl-cellulose precipitation (MCP) approach for the first time. In addition, a non-targeted metabolomics approach was applied to determine the most abundant phenolic classes present. Polymeric procyanidins were found to be the most abundant type of polyphenol in apple pomace extracts and were generally oligomeric in nature. Multivariate statistical analysis revealed that the ferric-reducing antioxidant power (FRAP) was most strongly correlated with the polymeric procyanidin concentration. Noting that polymeric procyanidins may not cross the cell layer to exert antioxidant activity in vivo, their presence in apple pomace extracts may therefore overestimate the FRAP. This work highlights the importance of polymeric procyanidins in the phenolic diversity of apple pomaces, and it is proposed that in future studies, rapid MCP assays may be used for their quantification.

## 1. Introduction

Apples have been widely studied for their health benefits, and contain various compounds that are thought to reduce the risk of cancer, cardiovascular disease, asthma, and obesity, as well as Alzheimer’s and other age-related degenerative disease [1,2,3]. The health-providing properties of apples have been shown to lie in their being a rich source of soluble and insoluble dietary fibre [1]. As a result, a recent research focus has been on the development of apple fibre supplements sourced from waste streams (pomace) in the juice and cider industries [4,5,6,7,8,9]. Further to dietary fibre, apples are known to be rich in polyphenols, which are found in most plants, but are enriched in certain fruits like apples and grapes [10]. Polyphenols serve in the body as antioxidant, antiproliferative, and cell-signalling phytochemicals [1,11]. The non-absorbable polyphenols bound to apple fibre are also thought to provide protection against obesity and may improve gut microbial health [2]. 

Apples have a diverse polyphenolic profile, which may, in part, confer the wide range of phytochemical activities reported for human health. Various studies have been conducted to determine the most abundant polyphenols in apples and their byproducts, and have generally focused on lower molecular weight phenolic compounds (≤600 g/mol), which are readily identified and characterised by standard chromatographic techniques, including LC-MS [12]. This has identified the most important classes of phenolic compounds in apples to be flavonoids, which include flavan-3-ols, flavonols, anthocyanins, and dihydrochalcones (phloridzin and phloretin), and phenolic acids, of which chlorogenic acid is the most abundant. Among the flavonoid class of compounds, higher molecular weight forms of flavan-3-ols exist, known as procyanidins. The dimeric forms are most commonly reported in apple studies [12], but it has long been known that higher degrees of polymerisation can occur, from oligomers to large polymers [13,14,15]. Indeed, it has been reported that procyanidins are, in fact, the most abundant polyphenol in apples, ranging from 69% to 82% in fresh apples [13]. Early in vivo work identified potential anti-cancer and anti-obesity effects of oligomeric and polymeric apple procyanidins [2,16,17]. A more detailed analysis of the structure–function relationships of apple procyanidins revealed remarkable differences in their potential anti-cancer activity using various in vitro assays [18]. 

Despite these early studies that identified the importance of polymeric procyanidins in terms of their concentration and impact on human health, much of the subsequent work on apple polyphenols has concentrated on phenolics of a lower molecular weight, including only the dimeric and, in some cases, trimeric forms of procyanidins [3,12,19]. Their exclusion from these studies may have been due to an early observation that polymeric procyanidins are poorly absorbed across the cell layer of the small intestine, thereby having limited intracellular impact [20]. Other work has shown that oligomeric procyanidins may be largely broken down into component monomers or dimers in the stomach [21], or further converted to phenolic acids by colonic microflora [20]. Therefore, although the downstream bioavailability of polymeric procyanidins may be dependent upon their in vivo conversion, their quantitative contribution to the overall apple polyphenol profile is nonetheless important. 

In an early study on polymeric procyanidins, the authors noted that while a multi-dimensional spectrum of phytochemical activities for apple procyanidins could be inferred, a lack of data on their concentrations in foods prevents clear conclusions on their in vivo role in human health being drawn [18]. Certain recent studies seeking to optimise the extraction of apple polyphenols have again highlighted the important contribution of polymeric flavan-3-ols [22,23]. However, it is noteworthy that data on polymeric flavan-3-ol concentrations and size (mean degree of polymerisation, mDP) in apple pomace remains scarce in the literature. To this end, we noted that a rapid, precipitation-based technique using methyl cellulose (MCP) to quantify polymeric proanthocyanidins, or condensed tannins, in grapes or wine [24] had not yet been applied in apple extracts or products. An opportunity was therefore present to screen apple materials for polymeric procyanidins, and to subsequently relate this to general phenolic composition and antioxidant potential.

Our selected focus was to characterise apple pomace, due to the increasing interest in its valorisation as a waste product of juice and cider production, for use as a dietary supplement [6,7,8,25,26]. The project aimed to conduct detailed compositional analysis of pomace fibre sourced from fresh apples or commercial processing, in order to determine the relative proportions of dietary components, including polyphenols. Further to polymeric procyanidins, a metabolomic approach was undertaken to semi-quantitatively determine the abundance of important lower molecular weight phenolic compounds. Multivariate statistics was then applied in order to explore differences in the polyphenolic composition between apple pomaces, and also to determine their relevance to antioxidant potential, measured as ferric-reducing antioxidant power (FRAP), which has been recommended for use in apple-derived products [27].

## 2. Results

### 2.1. General Compositional Characterisation of Apple Pomaces

The water content of the pomaces was between 69% and 80% with no clear pattern observed between the apple cultivars studied (results not shown). The pomace prepared from the commercially processed material had a 71% water content, and was therefore not substantially lower than that prepared from fresh apples. The composition of the pomaces prepared from fresh apple samples were compared on a dry weight basis (Table 1), since this was more relevant to the finished product (fibre and flour) from valorised apple processing waste. It was evident that sugars, in this case estimated from the quantification of fructose, glucose, and sucrose in the samples, formed the bulk of the material on a dry weight basis. The sugar content of the pomaces prepared from the fresh apples was between 46% and 69%, with higher sugar values being observed in the Fuji apple samples. The sugar content of the pomaces prepared from commercial juice processing (pomace) and which contained multiple (or unknown) apple cultivars had a somewhat lower sugar content than those from fresh apples, at 33–36%, which may reflect a greater extraction of sugar during the juice contact step.

Quantitatively, cell wall material, including lignin, pectin, hemicellulose, and cellulose, formed the greatest proportion of the dry material after sugars, at between 19% and 33%. Of the cell wall components, lignin formed the bulk of the material (9–20%), followed by pectin (5.6–10%), hemicellulose (2–4.1%), and cellulose (0.7–3%) in decreasing proportions. The estimated contributions of lignin and cellulose were different to that reported in the literature for apple pomaces, although direct comparison was complicated by the high contribution of free sugars and the presence of starch in our samples, which was not observed by others [28]. The relatively higher than expected contribution of lignin may have been due to the presence of non-extractable, covalently-bound phenolics [23], as these may have remained in the dry residue following hydrolysis. Furthermore, it is interesting to note that the commercial pomace material (Adelaide Hills) had undergone a pre-treatment with pectolytic enzymes during the juicing process, and it had a lower pectin content than other commercial apple pomace flours, but it was not necessarily lower than that observed in the pomaces prepared from fresh apples. These results indicate that irrespective of the apple processing conditions, pomaces retain a relatively consistent content of dietary fibre and may have broad application as a fibre supplement. As expected, starch was proportionally low in the pomace material and was generally less than 1%, with only one Fuji apple sample having a starch concentration of 2%. Protein was analysed in a subset of pomace samples and was between 2% and 4% of the dry weight.

### 2.2. Phenolic Composition of Apple Pomaces

A key focus of this study was to determine the variability and proportion of phenolics in apple pomaces, with a view to exploring their potential use as a phenolic-rich dietary supplement. While the composition of low molecular weight phenolics has been extensively researched in apple materials for their health benefits, the role of polymeric apple procyanidins have been the focus of relatively few studies [2,16,17,18,29]. For comparative purposes, the concentration of total phenolic compounds (monomeric and polymeric) and polymeric procyanidins was determined in epicatechin units (Table 1). The total ethanol-extractable phenolics in the apple pomaces was between 0.5% and 1%, while polymeric procyanidins ranged from not detected up to 0.7%. However, only one apple pomace sample had no detectable polymeric procyanidin (Royal Gala). The coefficient of variation determined for the range of samples analysed was far higher for polymeric procyanidins (71%) than total phenolics (36%). As a result, the proportion of polymeric procyanidins of total phenolics (as epicatechin units) could be as high as 59% (based on 280 nm absorbance in epicatechin units). The polymeric procyanidin composition was determined by phloroglucinolysis (Table 2) and, as expected, primarily consisted of epicatechin as both the terminal and extension subunits, with a minor contribution of catechin as a terminal subunit [15,30]. The mean degree of polymerisation (mDP) was ≤5, corresponding to a maximum molecular mass average of 1487 g/mol. Hence, polymeric procyanidins in the apple pomaces could be considered to be generally oligomeric molecules. As mentioned previously, there are limited reports of the polymeric procyanidin concentration in the literature, but our results are in agreement with others who reported a concentration of ≈0.3% and an mDP of 4.7 [23]. Although an investigation of the molecular mass distribution of apple procyanidins revealed that very high levels of polymerisation can exist, the mDP and associated molecular mass of commercial apple procyanidins is generally low relative to other fruits like grapes [15,31,32]. Only certain cider apple varieties have been shown to have procyanidins of a much higher molecular mass than that reported here [30,33]. For the apple samples for which multiple cultivar samples were analysed, there were no clear differences observed for total phenolics or polymeric procyanidin concentrations. This indicates that for the commercial apple cultivars studied, the environment may influence phenolic composition more significantly than the genotype. 

The anthocyanin concentration in pomaces showed a high coefficient of variation (112%), but very low concentrations, at between 0.01 and 0.9 mg/g of the dry weight (<0.1%). Although the anthocyanin content of the deeply pigmented variety Kanzi was substantially higher than the other apple samples, the relatively low anthocyanin concentrations in comparison to polymeric procyanidins may reflect the proportional dilution of skin with pulp material, as pomace is formed during juicing. 

Further characterisation of the profile of phenolic compounds (smaller than 600 g/mol) in the apple pomaces was conducted using a non-targeted LC-MS metabolomics approach. Of the compounds that could be putatively identified, the most abundant found in the apple samples are shown in Table 3. These were primarily flavonoids, the hydroxycinamic acid chlorogenic acid, and phenolic glycosides. The relative abundance of each compound was semi-quantitatively assessed as catechin equivalents and is provided as a concentration range (Table 3) and as Supplementary Materials Table S1. The estimated concentration of these lower molecular weight (≤610 g/mol) phenolics was low in comparison to the quantification of polymeric procyanidins and anthocyanin. Monomeric and dimeric flavan-3-ols, as well as flavonols, ranged from 3 to 22 µg/g. Chlorogenic acid was less than 14 µg/g, while phloretin was less than 2 µg/g. Importantly, the concentrations of native, identifiable polyphenols in the apple pomace extracts were far lower than expected, considering that the total phenolic content was between 0.5% and 1% w/w. This discrepancy is likely due to the susceptibility of the major monomeric apple polyphenols to oxidation by polyphenol oxidase during processing [34]. Therefore, after the polymeric procyanidins, which were proportionally the most significant polyphenol component, the presence of a substantial proportion of phenolic compounds that were neither identified nor quantified by LC-MS most likely reflects a conversion to oxidation products. However, it is noteworthy that research suggests that apple polymeric procyanidins may also be less extractable following oxidative processing due to the formation of covalent linkages with polysaccharides, and may have been excluded from the assays [23].

### 2.3. Measures of Antioxidant Potential in Apple Pomace Extracts

The antioxidant potential of the apple pomace extracts was also determined as the FRAP, as either FeSO_4_ or Trolox equivalents (Table 4). Although limitation exists in the in vivo relevance of various antioxidant potential assays [35], this approach was selected as it is widely applied and recommended for use in apples [27]. Furthermore, in this work, we coupled the assay with detailed phenolic compositional analysis, which has been recommended as an appropriate step to identifying the link between target compounds and antioxidant assays [35]. The FRAP measurement determined on a dry weight basis ranged from 20 to 85 µmol/g in Trolox equivalents and from 22 to 94 µmol/g in FeSO_4_ equivalents. The coefficient of variation in the FRAP for the dataset was 33%, similar to that observed for the total phenolics measurements. Moreover, as found for the phenolic compositional measures, no clear effect of cultivar was found in the FRAP analyses. In comparison with other studies on fresh apple and apple pomaces that have used a FRAP assay to estimate the antioxidant potential, a wide range of published values exist [11,27,36,37,38,39,40,41]. The FRAP values reported in this study were within the range of these other reports. However, these studies used different drying approaches (lyophilisation, oven-drying and sun-drying), methods of extraction (water, ethanolic, methanolic, and acetone), and standards of quantification, rendering the results only loosely comparable. Generally, pomace extracts that have undergone oxidation via processing or drying have a smaller FRAP range on a dry weight basis, at 3–37 µmol/g [8,37,39,40], when compared to skin and cortex extracts from fresh apples that have a higher upper threshold (1–190 µmol/g) [11,27,42]. The somewhat lower FRAP results we report here may reflect a combination of both sample variability and oxidative processing.

### 2.4. Multivariate Modeling of Apple Pomace Phenolic Composition

**Figure 1:**
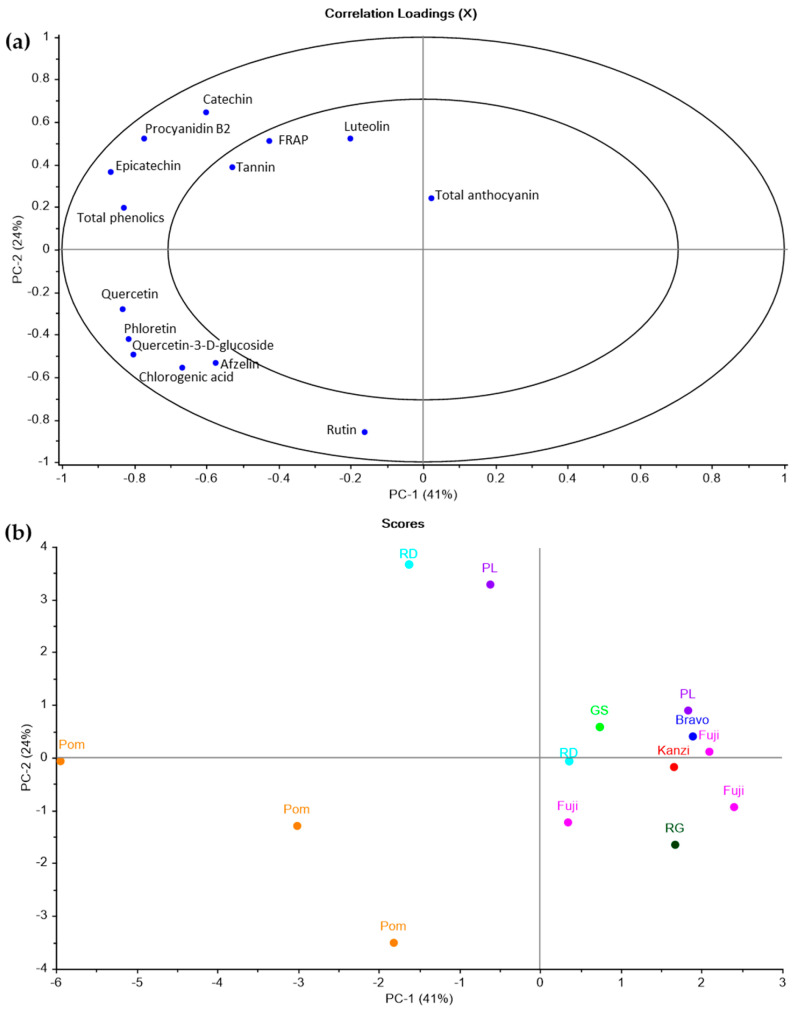
Principal component analysis results showing the variation in polymeric procyanidins (designated as “tannin”) and ferric-reducing antioxidant power (FRAP) with quantitative and semi-quantitative data generated for apple pomace samples. (**a**) Correlation loadings; (**b**) scores plot showing commercial pomace (Pom) and fresh apple pomaces designated as their cultivar names (RD, Red Delicious; PL, Pink Lady; RG, Royal Gala).

Further multivariate analysis to understand the relationships between apple pomace phenolic composition and FRAP was undertaken using PCA, using both quantitative and semi-quantitative phenolic data. The results are shown in Figure 1. The first two principal components (PCs) explained 65% of the X variance and 93% in five PCs. Inclusion of PC 3 and above did not show any further separation of the samples in terms of their compositional variability. From the correlation loadings plot, it was evident that separation of the commercial pomaces from the fresh apple pomaces occurred on both PCs 1 and 2. Commercial pomace samples were negatively loaded on both PCs, having proportionally higher levels of most phenolics, particularly chlorogenic acid, phloretin, and multiple flavonols. Higher levels of chlorogenic acid may reflect the inclusion of some core material in the pomace, as this apple component has higher concentrations of this compound [43], and since the cores were removed from the fresh apples prior to processing. The increased phloretin and quercetin may be due to release of the aglycone from the glycoside form with longer processing times. However, higher quercetin-glucoside concentrations suggest a greater contribution of skin material to the commercial pomaces [43], which may reflect differences in juice processing conditions. For the fresh apple pomace samples, no clear separation was observed in terms of compositional variability by PCA and were generally closely grouped in the scores plot (Figure 1b). Two fresh apple pomace samples were separated with high loadings on PC 2 relative to all other samples, and this was primarily due to higher levels of catechin, epicatechin, and procyanidin B2. 

An important observation was the correlation found between compositional variables evident from the PCA (Figure 1a). Antioxidant capacity, measured as the FRAP (Table 4), was found to be correlated with the total phenolics and polymeric procyanidin measures, as well as the flavan-3-ols catechin, epicatechin, and procyanidin B2. Surprisingly, other phenolics measures, including flavonols, phloretin, and chlorogenic acid, were negatively loaded on both PCs 1 and 2. A separation of the FRAP from these measures was evident on PC 2, suggesting a weaker relationship with the FRAP than was found for flavan-3-ols. This was studied further using PLSR, with the Royal Gala sample in which polymeric procyanidin was not detected, excluded from the model. All phenolic measures, including the procyanidin molecular mass determined by phloroglucinolyis, were initially included in the analysis to model the FRAP response. A subset of variables was identified as being significant via an uncertainty test, as well as by observed high correlation loadings, and the PLSR model developed using these is provided as Supplementary Materials Figure S1). It was found that when using this variable subset, an *R*^2^ of calibration of 0.81 was obtained, with an *R*^2^ validation of 0.61. The root mean square error (RMSE) of the prediction was 6.8 for the calibration and 10.5 for the validation. Maximal explained variance was achieved within three factors. 

In the PLSR model, the polymeric procyanidin measured by the MCP method was found to be most strongly correlated with the FRAP measure, with the highest positive weighted regression coefficient in the model of 0.42 (Supplementary Materials Table S2). Indeed, simple linear regression analysis of the polymeric procyanidin measure and the FRAP alone provided an R^2^ of 0.63 (result not shown; 0.62 including the Royal Gala sample). As found for the PCA, the flavan-3-ol class of compounds had high loadings on the first factor, yielding positive weighted regression coefficients for procyanidin B2 (0.15), epicatechin (0.13), and catechin (0.09). Although the procyanidin molecular mass and total anthocyanin measures were not strongly correlated with the concentration of polymeric procyanidins, these variables nonetheless had positive loadings on the second factor of the PLSR model, with weighted regression coefficients of 0.27 and 0.15, respectively. On the contrary, quercetin, quercetin-glucoside, phloretin, and chlorogenic acid were negatively loaded on factor 2 and, as a result, provided negative weighted regression coefficients in the model. These results were somewhat unexpected, given that the majority of research into the antioxidant properties of apple pomace have shown that quercetin and chlorogenic acid have a higher antioxidant capacity by the FRAP than other apple phenolics [44]. However, in that study, the FRAP measured for quercetin and chlorogenic acid was lower than that found for procyanidin B2, and polymeric procyanidins were not considered. The PLSR analysis showed, for the first time, that polymeric procyanidins require consideration for their contribution to the antioxidant potential in apple pomace, together with other phenolic compounds.

The oversight to include polymeric procyanidins as important contributors to the measure of antioxidant potential using various methods may indeed be due to the fact that molecules of a certain size and/or dimension are not transported intra-cellularly and therefore are expected to have no direct impact as antioxidants, despite the observed in vitro results [20]. Irrespective of this, the current results indicate that polymeric procyanidins may make a significant contribution to the measure of FRAP in apple pomace extracts, in turn suggesting that they may also contribute to other analytical measures of the antioxidant potential in apple [27] and possibly other plant or food sources. This highlights that the contribution of polymeric material should be quantitated and subsequently excluded from antioxidant assays when required, particularly as these compounds could cause confounding results when present in relatively high proportions compared to lower molecular weight phenolics. This is important, considering that other lower molecular weight apple phenolics were not significant contributors to the FRAP relative to polymeric procyanidins in the PLS model. 

Moreover, the results demonstrate that measures of antioxidant potential alone may not factor in the spectrum of activities present within the phenolic profile. Polymeric phenolic material may be broken down to monomeric intermediates by stomach acid [21], or acted upon by microflora in the colon to be converted into other beneficial intermediates at a later stage [20]. The work of our group to date demonstrates the importance of understanding the downstream bioconversion of polymeric procyanidins via other biological sources [45]. As a result, it is highlighted here that specific data on the role of polymeric procyanidins from apple pomace in health-related phenomena requires further definition.

## 3. Discussion

This study presented, for the first time in apple-based extracts, the use of a precipitation-based MCP approach to quantify polymeric procyanidins. This revealed that, in agreement with the findings of others, polymeric procyanidins are quantitatively the highest contributor to total phenolics in comparison to other categories of phenolic compounds [13,23]. It was shown that a wide range polymeric procyanidin values can be found in apple pomaces, up to 0.7% (*w*/*w*) of the dry weight and up to 59% of the total phenolics present. While this reflects a relatively low proportion of material in comparison to major contributors like sugars and fibre, it is nonetheless high in terms of nutritive phenolic value relative to other fruit sources, which are generally ≤0.8% (*w*/*w*) of the dry weight, with only one sample (Aronia berries) reported to be higher, at 1.7% [10]. The reason for this relative concentration of phenolics on a dry weight basis compared to whole fruit may be due to the proportional loss of juice, and thus a portion of sugar, in pomaces. Indeed, the average weight per apple in this study was 172 and 151 g for whole or cored apples, respectively. The expected dry weight yield of pomace for cored, juiced apples was between 4% and 10% for the samples included in this study, with an average yield of 8%. Relating this back to the fresh weight of apples, this indicates that only 12 g of pomace fibre would provide the phenolic equivalent of a whole apple. 

Moreover, the results revealed that polymeric procyanidins were correlated with the antioxidant potential of the pomace fibre extracts studied, as estimated by the FRAP method, which has not yet been reported for apple sources. Procyanidin B2 was putatively identified in this study, and then confirmed using an authentic standard. Procyanidin B2 and total anthocyanin were the most well-correlated with the FRAP in the predictive model after polymeric procyanidin. Procyanidin B2 has been implicated as a significant role-player in the FRAP and other measures of antioxidant potential in multiple studies [36,44,46,47]. The procyanidin B2 semi-quantitatively detected in our samples was expected to be within the range of 0.5 to 21 µg/g. Generally, procyanidin dimers are found in a similar concentration range to epicatechin [3], which, in this study, was semi-quantitatively determined to be between 3 and 19 µg/g. Polymeric procyanidins, on the contrary, were from 740 to 7040 µg/g where present in the apple pomace samples. Therefore, the substantial quantitative contribution of polymeric procyanidins to the antioxidant potential of apple pomace must be considered, in addition to the role of their structure. In relation to the polymeric procyanidin structure, it was noteworthy that the mDP was generally ≤5, corresponding to a maximum molecular mass of 1484 g/mol. This indicates that the apple procyanidins were generally oligomeric in nature. A fractionation study on cocoa procyanidins showed that the antioxidant activity measured as the FRAP and DPPH increased in a dimer-enriched fraction relative to catechin and epicatechin [48]. A further increase in antioxidant activity was found in a trimer-enriched fraction, but no change was observed between tetramer- and hexamer-enriched fractions [48]. These findings generally lend support to the observation that the polymeric procyanidin fraction may make a greater contribution to the antioxidant potential in apple phenolic extracts than previously thought. However, given that polymeric procyanidins are unlikely to have intracellular functionality [20], it could be considered that their presence may overestimate the antioxidant potential data generated in assays. While our results are particularly relevant to apple products, this could be considered for all antioxidant-rich products that contain polymeric procyanidin.

This study also highlighted the proportional contributions of sugars and fibre to apple pomace. The literature on apple pomace reports a wide range of reducing sugars in dry pomace from 29% to 62% for different cultivars [49,50,51], which is similar to what we observed here. Due to the high percentage of sugars, the relative proportion of dietary fibre was low. Reports that define the proportion in which apple fibres could potentially be incorporated into food products range from low, such as 3–5% in smoothies, bread, or yoghurt formulations [7,8,52], to as high as 25% in sweeter baked goods such as cookies [53]. Given the high proportion of sugar, this may limit the range of food-based applications for which it can be used, as it is effectively a sugar substitute. A key study demonstrated that apple pomace may be processed further using selected fungal innocula, decreasing the sugar content to 3% and doubling the protein [5]. While this bioconversion process may enrich the nutritive value of apple pomace, and thus diversify how it can be applied in food production, the impact of this process on phenolic composition and antioxidant potential remains unknown. Based on the results of our work, we propose that future research could concentrate on the development of processing technologies, which can enhance the nutritive and fibre content of apple pomace, while simultaneously retaining phenolic diversity and quantity. 

## 4. Materials and Methods

### 4.1. Preparation of Apple Pomace Samples

Three types of apple pomace samples were collected and analysed: Fresh apple pomaces prepared under laboratory conditions (11 samples), fresh commercially processed pomace (one sample), and dry commercial apple pomace flour (two samples). Fresh apples were sourced from three regions within South Australia (SA): The Barossa Valley, Adelaide Hills, and McLaren Vale. A total of seven cultivars were sampled. Replicate samples were obtained for the Fuji, Pink Lady, and Red Delicious cultivars. Fresh apples were selected so as to not have undergone wax treatment prior to processing, and were collected close to the time of harvest. The apples were sealed and stored at 4 °C for a maximum of two months prior to processing. The apples were then cored, sliced, and processed using a Breville 900 W Juice Fountain. Pomace (pulp and skins) from the juice processing was collected, mixed to ensure homogeneity, and a subsample was transferred to a 50 mL centrifuge tube and frozen at −80 °C. Frozen material was homogenised to a fine powder in liquid nitrogen with an electric grinder (IKA A11 basic, IKAWorks, Asia, Rawang, Malaysia). The powders were then weighed into centrifuge tubes, as required, and frozen at −80 °C until used. The fresh commercial apple pomace sample was obtained from Ceravolo Orchards in Ashton Hills, SA, following juice processing, and was noted by the producer to contain a combination of Pink Lady, Granny Smith, Royal Gala, Kanzi, and Red Delicious apple cultivars. During commercial processing, 1 kg of ascorbic acid and 200 mL of Novozymes Pectinex Ultra Olio enzyme (Novozymes, North Rocks, NSW, Australia) were applied per tonne of apples crushed. The enzyme contact period was up to 75 min at an ambient temperature (12–15 °C). Commercial pomace (pulp, skin, and seeds) was stored for 48 h at 4 °C and then frozen at −20 °C until used. A subsample of pomace was homogenised to a fine powder in liquid nitrogen with an electric grinder (IKA A11 basic, IKAWorks, Asia, Rawang, Malaysia), sampled, and stored at −80 °C as described above. To obtain a dry weight measure for apple pomace samples, 1 g of material was weighed into pre-tared containers and dried at 55 °C for 48 h, and the difference calculated. The two dried apple pomace materials were sourced from Packenham Upper, Victoria Australia (apple flour) and Illinois, USA (apple fibre) as reference samples for a compositional comparison of the commercial pomace sample with products already on the market. Once opened, these products were sealed and stored at 4 °C until analysis was completed. 

### 4.2. Analysis of the Ethanol-Soluble Fraction of Apple Pomace

A 1 g sample of frozen apple pomace powder from homogenate or 0.5 g of dried commercial apple pomace fibre was weighed into a pre-tared centrifuge tube and extracted in 10 mL of 70% *v*/*v* aqueous ethanol for 18 h on a rotary shaker, centrifuged for 10 min at 3273× *g*, and the supernatant retained. All samples were extracted in duplicate. Each pellet was re-extracted in 5 mL of 70% *v*/*v* aqueous ethanol for 1 h on a rotary shaker, centrifuged under the same conditions, and the supernatants combined. The pellet was retained, reconstituted in 2 mL of ultrapure water, frozen, and lyophilised for later analysis of cell wall composition. The supernatants were dried under a stream of nitrogen at 35 °C and reconstituted in 3 mL of 20% *v*/*v* aqueous ethanol, then stored at 4 °C to be analysed within one week. To obtain a measure of total sugars, a 50 µL aliquot of each sample was combined with 50 µL of 0.16 M HCl and heated at 90 °C for 15 min to hydrolyse the sucrose. The samples were then diluted five-fold with ultrapure water and the concentration of total sugars (sucrose and hexose) as glucose and fructose units were measured by HPLC using a Bio-Rad HPX-87H column as described previously [54]. The total phenolics, polymeric procyanidins, and anthocyanins in the ethanolic extracts were determined using a high-throughput spectrophotometric approach as described previously [24]. The total phenolics and polymeric procyanidins were quantified against an external calibration of a pure (-)-epicatechin standard (Merck Pty. Ltd., Merck KGaA, Darmstadt, Germany). The composition of polymeric procyanidins in each extract was determined following drying of an aliquot under a stream of nitrogen, reconstitution in pure methanol, and subsequent analysis by phloroglucinolysis [31] using the modifications described previously [32]. The FRAP of the extracts was determined using a microplate assay as described previously [55], with a 120 min reaction time [36], using Trolox or FeSO_4_ (Merck Pty. Ltd., Merck KGaA, Darmstadt, Germany) equivalents as the units of quantification.

### 4.3. Analysis of the Ethanol-Insoluble Fraction of Apple Pomace

The lyophilised pellets retained following extraction (as outlined in Section 4.2) were weighed to determine the dry weight recovery of ethanol-insoluble solids. Ten milligram portions of dry material were weighed into pre-tared 1.5 mL screw-cap centrifuge tubes for the analysis of starch (1 × 10 mg), pectin and hemicellose (1 × 10 mg), or cellulose and lignin (1 × 10 mg) content, respectively. For the determination of starch in the samples, a published protocol was used [56]. For starch hydrolysis, thermostable α-amylase and amyloglucosidase enzymes (Merck Pty. Ltd., Merck KGaA, Darmstadt, Germany) were used. Glucose in blank samples and hydrolysates was determined using an enzymatic test kit (Vintessential Laboratories Pty. Ltd., Dromana, VIC, Australia). 

For the determination of pectin (combined soluble and insoluble) and hemicellulose, 1 mL of 50 mM citrate buffer, pH 3.5, was prepared containing 0.2 µL/mL of Novozymes Pectinex Ultra Olio enzyme. The ethanol-insoluble material was suspended by gently inverting and tapping the base of the tube. The samples were then shaken on an orbital shaker at 23 °C for 1 h. Following this, the tubes were centrifuged at 16,000× *g* for 10 min, and the supernatant transferred to a 2 mL centrifuge tube. The pellets were then washed with 500 µL of ultrapure water, centrifuged again, and the supernatants combined. The supernatant was dried at room temperature in a vacuum centrifuge. The pellets retained from the pectolytic enzyme step were frozen and lyophilised. Dry samples were stored at −20 °C until analysed. Dried pellets and supernatants were suspended in 600 µL of 2 M trifluoroacetic acid (TFA) and hydrolysed for 3 h at 100 °C. The TFA hydrolysate was then dried at room temperature in a vacuum centrifuge and resuspended in ultrapure water. The TFA hydrolysates from pellets were centrifuged at 16,000× *g* for 10 min and the supernatant removed to a fresh 2 mL tube. The pellet was then re-extracted with 500 µL of ultrapure water, centrifuged, and the supernatant added to the TFA hydrolysate. The combined supernatants were dried at room temperature in a vacuum centrifuge and resuspended in ultrapure water as described above. Reconstituted hydrolysates were diluted as appropriate, ribose internal standard (Merck Pty. Ltd., Merck KGaA, Darmstadt, Germany) was added, and monosaccharide sugars were quantified by derivatisation and HPLC as described previously [57]. For the determination of cellulose and lignin, 100 µL of 12 M sulfuric acid was added to a further 10 mg sample, mixed gently, and left to stand at room temperature for 1 h. Thereafter, 1100 µL of ultrapure water was added, and the samples were hydrolysed for 3 h at 100 °C, as described above. The samples were then cooled on ice, centrifuged at 16,000× *g* for 10 min, and the supernatant recovered. The glucose concentration in the supernatants was determined using an enzymatic test kit (Vintessential Laboratories Pty. Ltd., Dromana, VIC, Australia). The quantity of cellulose in the samples was estimated as glucose equivalents following subtraction of the glucose quantified in the other fractions. The pellets were washed with ultrapure water, frozen, lyophilised, and then weighed to obtain a gravimetric estimate of the lignin content. 

### 4.4. Protein Analysis

For analysis of the protein content in the pomace fibre powders, a subset of samples were selected. Frozen pomace samples were dried at 55 °C for 48 h and milled to a fine powder at 10,000 rpm for 1 min in a Retsch Grindomix GM200 (Retsch GmbH & Co., Haan, Germany). The apple powder was then sealed and stored at 4 °C until analysed. Commercial pomace flours were analysed directly. Powdered samples were sent to an analytical facility (Navlabs, Laverton North, VIC, Australia) for the determination of protein by the Kjeldahl method. Protein was estimated as the quantity of nitrogen multiplied by a factor of 6.25. 

### 4.5. Non-Targeted Phenolic Profiling by LC-MS

Freeze-dried homogenate or dried commercial apple pomace flour (1.0 g) was weighed into a 50 mL falcon tube and 10 mL of solvent mixture (2 mL of water, 4 mL of methanol, and 4 mL of chloroform) was added. The tubes were vortexed for 1 min and shaken in an orbital shaker (15 min at room temperature), then centrifuged for 25 min at 4000× *g* at 4 °C. The upper aqueous phase was collected, and the extraction was repeated on the lower phase by adding 6 mL of water/methanol at 1:2 *v*/*v*. The samples were vortexed (1 min) and re-extracted on the orbital shaker for 15 min. The samples were centrifuged and the upper phase was combined with the first extract. The combined upper phases were dried under a stream of nitrogen at 40 °C. The dried samples were reconstituted in 8 mL of ultrapure water. Phenomenex Strata-X polymeric RP SPE cartridges (Phenomenex, Lane Cove, NSW, Australia) were conditioned with 1 mL of methanol followed by 1 mL of ultrapure water. A 8 mL aliquot of each sample was loaded, and the cartridges were washed with 2% methanol (1 mL) and dried under vacuum for 5 min. The collected solvent was discarded and the phenolic fraction eluted with 1 mL of pure methanol. The extracts were dried under a stream of nitrogen for 15 min at 30 °C and resuspended in 75 µL of solvent A (0.5% methanol and 0.1% formic acid in ultrapure water) and 25 µL of solvent B (40% acetonitrile, 2% ultrapure water and 0.1% formic acid in methanol). The samples were transferred into 250 µL inserts in 2 mL HPLC vials for LC-MS analysis. Prior to injection, each sample was spiked with a solution of morin internal standard (Merck Pty. Ltd., Merck KGaA, Darmstadt, Germany) to a final concentration of 50 mg/L. Separation was performed on a Thermo Vanquish Horizon coupled to a Thermo Orbitrap IDX High-Resolution Fourier Transform mass spectrometer (HRFT-MS) (Thermo Fischer Scientific, Adelaide, SA, Australia). For HPLC, sample aliquots (2 μL to MS1 and 4 μL to MS-2) were injected to a Phenomenex Kinetex F5 (2.6 µm, 150 × 2.1 mm) column, at an initial flow rate of 0.4 mL/min with 100% solvent A. Solvent B was varied throughout a 42 min run to 1% at 6.25 min, 7.5% at 20 min, 60% at 30 min, and 90% at 33 min until 38 min. From 38 to 39 min, the solvent ratio oscillated from 100% solvent A to 90% solvent B every 0.2 min. After 39 min, the solvent was held at 100% A until 42 min. The HPLC temperature was set at 30 °C. For MS, the samples were acquired in MS-ESI negative mode. The MS was operated at an ion transfer tube temperature of 275 °C and a vaporiser temperature of 300 °C with a spray voltage of 3400 V. The final number of molecular features detected was 105. The term “molecular feature” describes a two-dimensional bounded signal: A chromatographic peak (retention time) and a mass spectral peak (*m*/*z*). Analysis was performed by the AWRI’s Metabolomics Australia node, as described in Section 4.6.

### 4.6. Molecular Feature Extraction

Following LC-MS, Thermo Compound Discoverer Software (Version 3.1) was used to perform molecular feature extraction and feature grouping into compounds. The term molecular feature describes a two-dimensional bounded signal: A chromatographic peak (retention time) and a mass spectral peak (*m*/*z*). MS1 signals were acquired for the sample replicates and a pooled mix of replicates was used to monitor the instrument performance along the sequence. The raw data matrix was submitted to a normalisation step, which consisted of applying median normalisation to correct for any possible inter-run instrument variability. Following this, the coefficient of variation (CV%) of the pooled mix of replicates was calculated and compounds with a CV% value above 20 were removed from the data matrix. Following this, the total number of reliably detected features was 259. Library matching of the MS1 and MS2 spectra was performed using the internal AWRI database and the mzCloud database. For compounds where no putative identification could be assigned, molecular formulas were provided. A summary of the putatively identified compounds and/or assigned formula, together with their annotation details and instrumental response (median normalised chromatographic areas) for each sample, is reported as Supplementary Materials Table S3. A subset of phenolic compounds were found to be the most abundant in the apple pomace samples. In order to evaluate their relative abundance between samples, the amount of each compound was expressed semi-quantitatively using a catechin (Merck Pty. Ltd., Merck KGaA, Darmstadt, Germany) calibration curve normalised against the morin internal standard. Procyanidin B2, putatively identified as an unspecified procyanidin dimer by mass spectrometry, was confirmed using an authentic standard (Merck Pty. Ltd., Merck KGaA, Darmstadt, Germany). 

### 4.7. Multivariate Statistical Analysis

Principal component analysis (PCA) and partial least squares regression (PLSR) were performed using the Unscrambler 11.0 (CAMO Analytics, Aspen Technology, Bedford, MA, USA). Data were scaled as the inverse of the standard deviation for multivariate analyses. PLSR analysis included an uncertainty test to identify significant variables in addition to the correlation loadings. All PCA and PLSR analyses were performed with full cross-validation.

## Figures and Tables

**Table 1:** Composition of apple pomaces prepared from fresh apples (cultivar name) or commercial processing (pomace, multiple cultivars) presented on a dry weight basis (mean ± standard error).

Apple Pomace	Source ^2^	Sugars (mg/g D.wt)	Total Phenolics (mg/g D.wt) ^3^	Anthocyanin (mg/g D.wt)	Polymeric Procyanidin (mg/g D.wt) ^3^	Pectin (mg/g D.wt)	Hemicellulose (mg/g D.wt)	Cellulose (mg/g D.wt)	Lignin (mg/g D.wt)	Starch (mg/g D.wt)	Protein (mg/g D.wt) ^4^
Red Delicious	Barossa Valley, SA	524.7 ± 14	5.63 ± 0.03	0.13 ± 0.03	2.52 ± 0.17	66.89 ± 1.05	29.52 ± 0.16	14.72 ± 3.21	113.26 ± 2.00	5.59 ± 0.31	
Red Delicious	Adelaide Hills, SA	482.6 ± 7	12.02 ± 0.08	0.32 ± 0.02	7.04 ± 0.19	60.82 ± 0.11	30.14 ± 0.31	18.98 ± 3.97	164.71 ± 3.87	1.41 ± 0.08	
Fuji	Barossa Valley, SA	688.9 ± 13	4.98 ± 0.15	0.07 ± 0.01	2.78 ± 0.12	44.26 ± 4.05	19.76 ± 0.27	8.04 ± 1.09	165.17 ± 1.01	1.11 ± 0.30	
Fuji	Barossa Valley, SA	539.3 ± 6	3.31 ± 0.02	0.04 ± 0.02	0.94 ± 0.14	72.49 ± 1.22	28.25 ± 0.98	21.64 ± 3.96	111.59 ± 4.69	1.99 ± 0.70	19.0
Fuji	McLaren Vale, SA	557.4 ± 16	7.07 ± 0.07	0.15 ± 0.03	3.27 ± 0.15	64.98 ± 2.83	41.44 ± 0.84	6.99 ± 1.80	88.30 ± 2.07	21.13 ± 0.96	
Pink Lady	Barossa Valley, SA	464.8 ± 19	5.83 ± 0.02	0.10 ± 0.00	1.27 ± 0.05	82.53 ± 0.78	30.88 ± 0.15	19.87 ± 4.92	103.57 ± 9.72	5.53 ± 1.86	19.0
Pink Lady	Adelaide Hills, SA	543.9 ± 10	7.85 ± 0.45	0.25 ± 0.02	2.91 ± 0.19	55.81 ± 3.49	24.18 ± 2.84	9.94 ± 1.80	99.29 ± 10.09	2.10 ± 0.13	
Royal Gala	Adelaide Hills, SA	521.8 ± 1	5.77 ± 0.10	0.13 ± 0.01	n.d.	73.06 ± 1.22	31.80 ± 0.01	18.40 ± 4.19	159.52 ± 4.33	3.35 ± 0.46	
Bravo	Adelaide Hills, SA	517.9 ± 14	8.12 ± 0.19	0.88 ± 0.03	0.86 ± 0.00	67.77 ± 1.58	26.12 ± 0.12	10.02 ± 0.32	115.31 ± 2.68	4.24 ± 0.11	
Kanzi	Adelaide Hills, SA	498.3 ± 8	4.92 ± 0.05	0.07 ± 0.02	0.74 ± 0.05	69.27 ± 4.88	31.68 ± 1.62	32.75 ± 1.20	106.32 ± 11.25	1.42 ± 0.37	
Granny Smith	McLaren Vale, SA	460.3 ± 4	6.10 ± 0.21	0.05 ± 0.03	2.54 ± 0.14	86.76 ± 2.14	36.01 ± 0.97	11.16 ± 0.22	137.79 ± 0.00	2.75 ± 0.81	
Pomace	Adelaide Hills, SA	360.1 ± 1	8.47 ± 0.35	0.18 ± 0.02	3.51 ± 0.34	60.87 ± 3.10	41.06 ± 0.28	9.47 ± 1.01	208.00 ± 3.33	2.87 ± 0.06	43.0
Pomace ^1^	Packenham Upper, VIC	334.7 ± 17	10.15 ± 0.22	0.15 ± 0.00	3.55 ± 0.02	89.63 ± 0.21	37.82 ± 0.62	11.24 ± 3.16	188.62 ± 15.81	2.88 ± 1.09	42.0
Pomace ^1^	Illinois, USA	328.1 ± 13	11.82 ± 0.17	0.16 ± 0.00	2.61 ± 0.22	101.96 ± 1.12	26.85 ± 5.22	19.27 ± 1.29	133.42 ± 32.53	9.41 ± 2.46	32.0

^1^ Commercial dry apple fibre product; ^2^ Apples and pomace were from Australia (SA, South Australia; VIC, Victoria) unless specified; ^3^ As epicatechin equivalents; ^4^ Protein analysis determined in a commercial laboratory by the Kjeldahl method on a sub-set of samples; n.d. = not detected; research laboratory analyses were conducted on duplicate samples.

**Table 2:** Polymeric procyanidin composition of apple pomace extracts prepared from fresh apples (cultivar name) or from commercial processing (pomace, multiple cultivars) determined by phloroglucinolysis.

Apple Pomace	Source ^2^	MM ^3^ (g/mol)	mDP ^4^	EC-ext ^5^ (mol%)	EC-ter ^5^ (mol%)	CAT-ter ^5^ (mol%)
Red Delicious	Barossa Valley, SA	1289 ± 23	4.44 ± 0.08	77.49 ± 0.39	18.21 ± 0.52	4.29 ± 0.13
Red Delicious	Adelaide Hills, SA	1179 ± 4	4.06 ± 0.02	75.40 ± 0.09	19.80 ± 0.08	4.79 ± 0.17
Fuji	Barossa Valley, SA	903 ± 109	3.11 ± 0.38	67.38 ± 3.94	24.31 ± 3.46	8.29 ± 3.94
Fuji	Barossa Valley, SA	970 ± 27	3.34 ± 0.09	70.06 ± 0.84	22.74 ± 0.93	7.18 ± 0.84
Fuji	McLaren Vale, SA	740 ± 15	2.54 ± 0.05	60.75 ± 0.80	22.00 ± 3.24	17.23 ± 0.80
Pink Lady	Barossa Valley, SA	1211 ± 59	4.17 ± 0.20	75.99 ± 1.17	21.06 ± 1.71	2.94 ± 1.17
Pink Lady	Adelaide Hills, SA	933 ± 3	3.21 ± 0.01	68.90 ± 0.09	24.38 ± 0.68	6.70 ± 0.09
Royal Gala	Adelaide Hills, SA	n.d.	n.d.	n.d.	n.d.	n.d.
Bravo	Adelaide Hills, SA	1484 ± 5	5.11 ± 0.02	80.44 ± 0.06	16.12 ± 0.27	3.43 ± 0.06
Kanty	Adelaide Hills, SA	933 ± 10	3.21 ± 0.04	68.91 ± 0.34	22.56 ± 0.17	8.52 ± 0.34
Granny Smith	McLaren Vale, SA	1487 ± 77	5.12 ± 0.27	80.43 ± 1.02	14.98 ± 0.66	4.57 ± 1.02
Pomace	Adelaide Hills, SA	1408 ± 167	4.75 ± 0.57	74.89 ± 2.61	17.46 ± 1.07	3.85 ± 2.61
Pomace ^1^	Packenham Upper, VIC	1050 ± 37	3.61 ± 0.13	72.33 ± 0.98	25.33 ± 0.89	2.32 ± 0.08
Pomace ^1^	Illinois, USA	816 ± 35	2.81 ± 0.12	64.38 ± 1.51	28.67 ± 0.21	6.94 ± 1.51

^1^ Commercial dry apple fibre product; ^2^ Apples and pomace were from Australia (SA, South Australia; VIC, Victoria) unless specified; ^3^ Polymeric procyanidin molecular mass (MM) based on subunit composition; ^4^ Polymeric procyanidin mean degree of polymerisation (mDP) based on subunit composition; ^5^ Molar percentage of epicatechin (EC) and catechin (CAT) as extension (ext) units or EC terminal (ter) units; n.d. = not detected; analyses were conducted on duplicate samples.

**Table 3:** Abundant phenolics putatively identified in apple pomace extracts by non-targeted LC-MS profiling, with their semi-quantitative concentration range as µg/g D.wt. in catechin units.

ID ^1^	Name	Formula	Molecular Weight (g·mol^−1^)	Retention Time (min)	Concentration Range (µg/g D.wt; Semi-Quantitative)
27	Catechin ^2^	C15 H14 O6	290.08	13.05	0.08–3.01
45	Chlorogenic acid	C16 H18 O9	354.10	17.07	0.60–14.35
63	Procyanidin B2 ^2^	C30 H26 O12	578.14	18.93	0.44–21.20
73	Phenethyl-primeveroside	C19 H28 O10	416.17	19.89	0.14–2.29
87	Epicatechin	C15 H14 O6	290.08	21.30	1.70–19.09
156	Quercetin-3-D-glucoside	C21 H20 O12	464.10	25.59	2.18–11.38
159	Rutin	C27 H30 O16	610.15	25.67	0.08–2.96
170	Phloretin	C15 H14 O5	274.08	26.22	0.02–2.12
190	Afzelin	C21 H20 O10	432.11	27.17	0.04–1.30
192	Quercetin	C15 H10 O7	302.04	27.87	0.03–6.93
195	Luteolin	C15 H10 O6	286.05	28.84	0.14–0.43

^1^ Molecular feature ID number as provided in Supplementary Materials Table S1. ^2^ Putatively identified by mass spectrometry then confirmed using authentic standard.

**Table 4:** Ferric-reducing ability of plasma determined in apple pomace extracts prepared from fresh apples (cultivar name) or from commercial processing (pomace, multiple cultivars).

Apple Pomace	Source ^2^	FRAP (µmol Trolox eq./g D.wt) ^3^	FRAP (µmol FeSO_4_ eq./g D.wt) ^4^
Red Delicious	Barossa Valley, SA	34.36 ± 0.41	38.64 ± 0.46
Red Delicious	Adelaide Hills, SA	85.25 ± 0.57	95.87 ± 0.64
Fuji	Barossa Valley, SA	55.07 ± 0.89	61.93 ± 1.00
Fuji	Barossa Valley, SA	20.17 ± 0.25	22.68 ± 0.28
Fuji	McLaren Vale, SA	40.70 ± 0.42	45.78 ± 0.48
Pink Lady	Barossa Valley, SA	41.51 ± 1.37	46.68 ± 1.54
Pink Lady	Adelaide Hills, SA	53.78 ± 3.36	60.47 ± 3.78
Royal Gala	Adelaide Hills, SA	37.67 ± 3.73	42.38 ± 4.19
Bravo	Adelaide Hills, SA	48.46 ± 0.14	54.50 ± 0.16
Kanzi	Adelaide Hills, SA	36.90 ± 0.30	41.50 ± 0.33
Granny Smith	McLaren Vale, SA	53.63 ± 3.09	60.33 ± 3.47
Pomace	Adelaide Hills, SA	68.76 ± 2.21	77.33 ± 2.49
Pomace ^1^	Packenham Upper, VIC	44.55 ± 0.50	50.11 ± 0.56
Pomace ^1^	Illinois, USA	45.63 ± 0.91	51.32 ± 1.02

^1^ Commercial dry apple fibre product; ^2^ Apples and pomace were from Australia (SA, South Australia; VIC, Victoria) unless specified; ^3^ FRAP, Ferric Reducing Ability of Plasma as Trolox equivalents; ^4^ FRAP, Ferric Reducing Ability of Plasma as ferrous sulfate equivalents; research laboratory analyses were conducted on duplicate samples.

## Data Availability

All data are provided in the article or as Supplementary Materials.

## Supplementary Materials

The following supporting information can be downloaded at: https://www.mdpi.com/article/10.3390/molecules28145494/s1, Table S1, Abundant phenolic compounds putatively identified in apple pomaces; Figure S1, Partial least squares regression analysis results for the prediction of the ferric-reducing antioxidant power (FRAP) as the Y-variable from a subset of significant phenolic apple phenolic variables; Table S2, Weighted regression coefficients from partial least squares regression analysis. Supplementary Table S3, Curated data matrix of normalised MS1 signals for putatively identified compounds in apple pomace extracts.

## Sample Availability

Samples of the compounds are not available from the authors.
