# Supplementary material for: Apple Pomace Compositional Data Highlighting the Proportional Contribution of Polymeric Procyanidins
Source: Molecules. 2023 Jul 18;28(14):5494. doi: 10.3390/molecules28145494 (PMC10384618; doi:10.3390/molecules28145494)
Supplement: Supplementary file 1 [file molecules-28-05494-s001.zip › molecules-2304552-supplementary.pdf]

Table S1. Abundant phenolic compounds identified in apple pomaces prepared from fresh apples (cultivar name) or commercial processing (pomace, multiple cultivars) using non-targeted LC-MS. Data is normalised to catechin equivalents as μg/g dry weight.

Apple pomace	Source[2]	Catechin	Epicatechin	Procyanidin B2[3]	Chlorogenic acid	Phloretin	Quercetin-3-D-glucoside	Quercetin	Rutin	Afzelin	Luteolin	Phenethyl-primeveroside
Red Delicious	Barossa Valley, SA	0.46	5.33	10.35	0.92	1.12	5.25	1.73	1.32	0.32	0.34	1.39
Red Delicious	Adelaide Hills, SA	1.73	11.92	16.03	n.d.	0.58	3.73	0.08	0.58	n.d.	0.31	0.78
Fuji	Barossa Valley, SA	n.d.	2.65	0.44	1.40	0.11	3.77	0.03	1.17	0.11	0.28	0.23
Fuji	Barossa Valley, SA	0.27	4.75	2.52	3.06	0.14	3.58	0.04	1.91	0.18	0.31	0.36
Fuji	McLaren Vale, SA	0.10	5.70	3.04	1.87	0.18	6.08	0.21	2.84	1.24	0.36	0.78
Pink Lady	Barossa Valley, SA	0.50	5.38	7.04	3.09	0.02	2.18	0.11	0.67	0.04	0.33	0.14
Pink Lady	Adelaide Hills, SA	3.01	13.12	14.75	2.42	0.23	3.83	0.10	0.08	0.04	0.37	0.51
Royal Gala	Adelaide Hills, SA	0.15	2.96	0.49	6.29	0.27	6.59	0.20	2.15	0.08	0.26	1.14
Bravo	Adelaide Hills, SA	0.08	1.70	1.33	3.10	0.06	4.89	0.20	0.74	n.d.	0.27	0.49
Kanzi	Adelaide Hills, SA	0.92	5.69	5.27	4.51	n.d.	2.86	0.08	2.00	0.23	0.33	0.20
Granny Smith	McLaren Vale, SA	0.97	7.58	10.52	0.60	0.14	3.50	0.17	1.82	0.44	0.28	0.72
Pomace	Adelaide Hills, SA	0.74	13.80	14.93	7.40	1.54	8.83	1.58	2.96	1.30	0.26	1.70
Pomace[1]	Packenham Upper, VIC	0.13	4.42	1.32	12.44	2.12	8.25	3.03	2.88	0.74	0.14	2.29
Pomace[1]	Illinois, USA	2.52	19.09	21.20	14.35	1.80	11.38	6.93	1.50	0.83	0.43	0.65

[1]Commercial dry apple fibre product; [2]Apples and pomace were from Australia (SA, South Australia; VIC, Victoria) unless specified; [3]Putatively identified as the dimer, then confirmed by mass spectrometry using the authentic standard.

Figure S1. Partial least squares regression analysis results for the prediction of ferric-reducing antioxidant power (FRAP) as the Y-variable from a sub-set of significant phenolic variables as the X-variables, in apple pomaces where polymeric procyanidins are designated as 'tannin'. (**a**) Correlation loadings; (**b**) Scores plot showing commercial pomace (Pom) and fresh apple pomaces designated as their cultivar names (RD, Red Delicious; PL, Pink Lady)

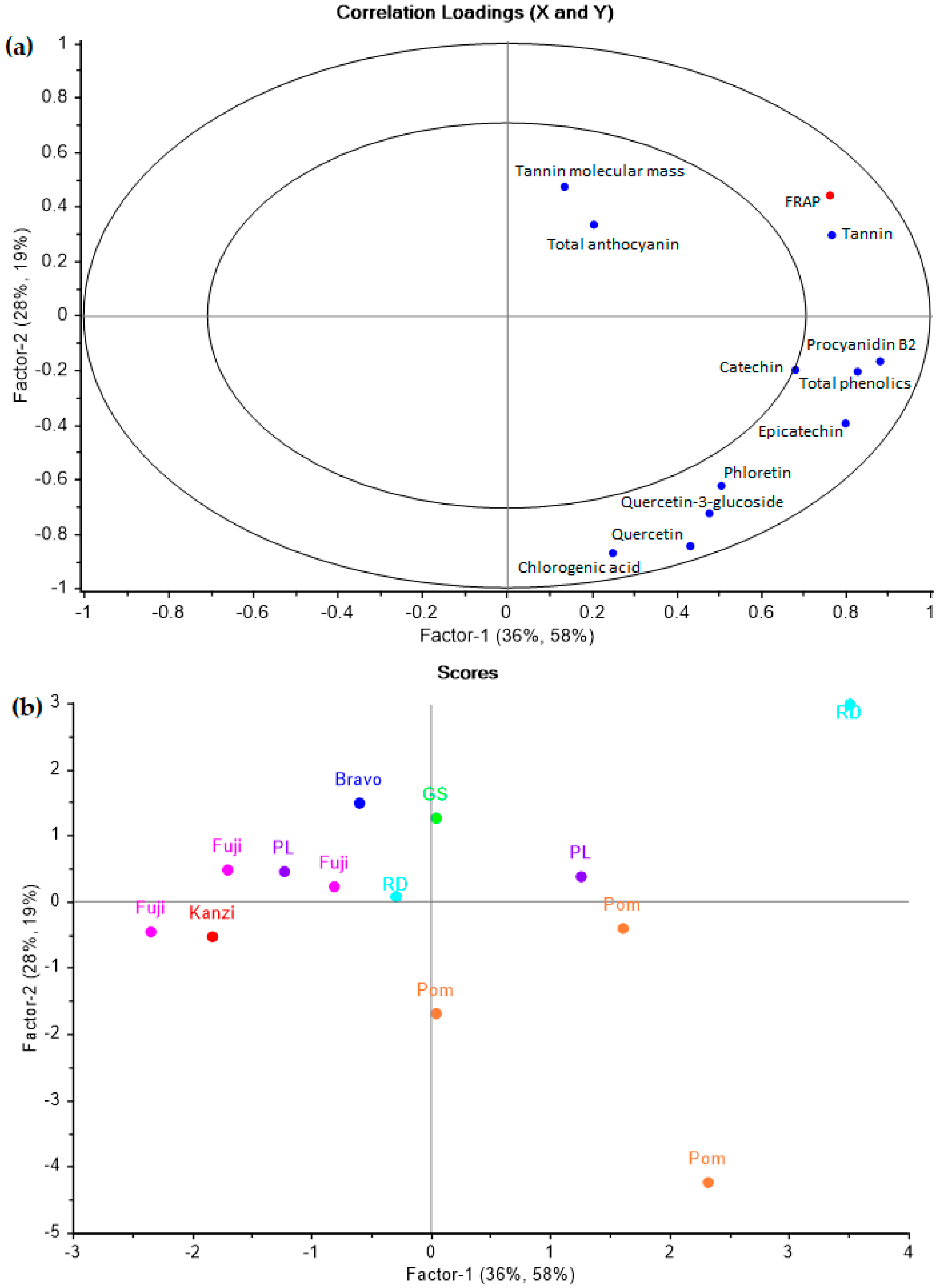

.

Table S2. Weighted regression coefficients from Partial Least Squares Regression analysis for the prediction of Ferric-reducing Antioxidant Power of Plasma (FRAP) from phenolic composition in apple pomace samples, where polymeric procyanidins are designated as 'tannin'.

	Weighted regression coefficient
Total phenolics	0.27
Total anthocyanin	0.15
Tannin	0.42
Tannin molecular mass	0.20
Catechin	0.09
Epicatechin	0.13
Procyanidin B2	0.15
Chlorogenic acid	-0.15
Phloretin	-0.02
Quercetin-3-D-glucoside	-0.05
Quercetin	-0.16

Table S3. Curated data matrix for molecular feature extraction by mass-spectrometry, following two steps of normalisation.

ID	Name	Formula	Molecular Weight	RT [min]	Barossa_RedDelicious	Adelaide Hills_Red Delicious	Barossa_Fuji1	Barossa_Fuji2	MclarenVale_Fuji	Barossa_PinkLady	Adelaide Hills_PinkLady	Adelaide Hills_RoyalGala	Adelaide Hills_Bravo	Adelaide Hills_Kanzy	McLaren Vale_GrannySmith	Adelaide Hills_Pomace	Commercial_Fibre_1	Commercial_Fibre_2	MMIX_R1	MMIX_R2	MMIX_R3	MMIX_R4	MMIX_R5	MMIX_R6	MMIX_R7	MMIX_R8
1	2-C-methylerythritol 4-phosphate	C5 H13 O7 P	216.04024	0.911	9.63	6.61	11.62	2.67	5.72	2.71	6.22	8.17	5.44	5.29	8.62	19.08	20.26	14.20	3.64	3.76	4.09	4.27	3.11	4.12	3.27	3.38
2	α,α-Trehalose	C12 H22 O11	342.11656	0.917	59.61	16.48	59.14	26.58	39.88	25.57	34.93	77.74	26.14	55.50	71.47	43.76	46.84	80.76	17.47	17.66	19.48	19.65	17.20	20.69	16.41	14.65
3	alpha-D-Glucopyranosyl 2-O-(2-methylbutanoyl)-alpha-D-glucopyranoside	C17 H30 O12	472.17957	7.014	1.10	0.02	2.59	2.99	6.21	16.98	28.22	0.24	0.42	32.94	0.01	16.56	11.31	1.07	4.89	4.20	4.92	4.40	3.44	4.07	3.30	3.32
4	α-D-Glucopyranosyl 3-O-(2-methylbutanoyl)-α-D-glucopyranoside	C17 H30 O12	426.17408	7.026	1.23	0.03	2.89	3.37	7.01	19.23	32.31	0.28	0.47	37.46	0.01	18.80	12.75	1.19	5.53	4.74	5.57	4.99	3.90	4.62	3.73	3.76
5	Catalposide	C22 H26 O12	482.14273	8.36	14.76	32.49	17.96	15.75	26.05	0.86	10.35	14.22	5.76	4.06	7.88	3.28	0.09	0.14	4.57	4.28	5.08	5.00	3.94	5.02	4.14	4.27
6	Mirabegron	C21 H24 N4 O2 S	396.16363	8.888	3.68	1.77	4.52	4.20	4.41	6.78	48.25	5.79	10.59	11.65	0.55	13.45	24.14	12.28	5.99	5.06	5.91	5.46	4.27	4.88	3.99	4.12
9	1-O-vanilloyl-beta-D-glucose	C14 H18 O9	330.09525	8.918	2.84	0.85	1.30	0.39	3.27	0.09	0.56	2.25	1.31	2.90	0.16	48.90	24.19	26.72	3.85	3.47	3.61	3.23	2.54	2.77	2.61	2.23
11	4-Acetyl-3-hydroxy-5-methylphenyl β-D-glucopyranoside	C15 H20 O8	328.11606	9.243	5.97	1.01	7.10	9.41	8.67	1.23	4.18	4.38	1.66	5.98	16.44	18.17	27.88	10.02	2.81	2.59	3.07	2.90	2.72	3.14	2.76	2.67
13	3-[3-(beta-D-Glucopyranosyloxy)-2-hydroxyphenyl]propanoic acid	C15 H20 O9	344.11124	10.422	5.62	1.10	9.68	7.09	8.67	4.71	4.73	16.13	4.65	8.57	8.97	43.96	53.93	34.02	5.14	4.58	5.36	5.07	4.06	5.18	4.14	4.19
14	Melilotoside	C15 H18 O8	326.10043	10.425	3.11	0.17	6.17	3.08	3.14	2.80	1.94	12.61	2.94	5.66	8.08	49.53	55.85	20.37	3.60	3.16	3.66	3.47	2.78	3.60	2.90	2.98
15	Mirabegron	C21 H24 N4 O2 S	396.16371	10.766	0.69	0.23	0.04	0.76	0.66	1.83	73.29	1.84	3.30	29.93	0.32	12.32	4.30	3.78	6.14	5.27	6.17	5.45	4.13	4.85	3.95	3.98
17	1-O-vanilloyl-beta-D-glucose	C14 H18 O9	330.09527	11.383	4.19	4.67	6.63	6.29	10.18	2.42	7.88	8.66	3.07	4.82	3.86	18.47	19.48	20.95	3.32	2.95	3.41	3.19	2.50	3.19	2.37	2.66
21	D(-)-Amygdalin	C20 H27 N O11	457.15911	12.296	45.93	0.00	0.01	0.03	0.03	0.02	0.02	0.01	0.01	0.03	0.01	30.67	154.62	339.75	8.68	8.38	8.80	9.77	7.50	10.12	7.43	6.71
22	alpha-D-Glucopyranosyl 2-O-(2-methylbutanoyl)-alpha-D-glucopyranoside	C17 H30 O12	472.17971	12.313	3.71	0.58	0.89	0.72	1.87	1.40	25.84	6.81	7.60	4.88	0.09	2.65	10.06	8.52	2.93	2.88	2.49	2.49	2.87	3.57	2.09	1.70
23	α-D-Glucopyranosyl 3-O-(2-methylbutanoyl)-α-D-glucopyranoside	C17 H30 O12	426.17424	12.319	7.94	1.84	4.03	4.74	8.24	3.38	48.65	14.55	14.89	8.79	0.22	5.77	22.75	16.00	5.15	5.86	5.49	5.38	5.09	6.77	4.90	3.95
27	Catechin	C15 H14 O6	290.07931	13.05	21.82	70.91	6.02	15.73	10.46	26.26	131.67	12.74	10.47	36.56	38.64	39.62	13.00	117.82	19.23	18.08	19.44	17.49	15.33	16.82	12.68	13.67
28	4-Allyl-2-methoxyphenyl 6-O-beta-D-xylopyranosyl-beta-D-glucopyranoside	C21 H30 O11	458.17937	13.183	7.06	0.50	10.96	10.27	8.49	7.81	9.10	29.12	11.95	3.17	12.26	54.98	85.62	53.48	6.04	5.79	6.45	7.45	5.14	5.38	6.00	5.08
32	Sobuzoxane	C22 H34 N4 O10	514.22648	13.728	8.84	6.57	27.32	13.66	5.56	0.92	5.01	5.66	3.00	18.73	4.38	8.04	21.11	3.10	2.81	2.55	3.23	3.29	2.71	3.54	3.26	3.25
40	1-O-vanilloyl-beta-D-glucose	C14 H18 O9	330.09526	15.992	6.45	8.02	3.71	4.17	5.73	3.46	7.01	7.88	6.92	4.21	3.39	12.88	10.09	13.23	2.93	2.62	3.10	2.97	2.36	2.91	2.36	2.07
42	Sobuzoxane	C22 H34 N4 O10	514.22663	16.372	4.87	0.43	2.84	7.67	10.11	25.80	13.07	1.34	4.10	19.39	2.99	13.94	10.90	6.40	4.95	4.21	4.87	4.47	3.53	4.10	3.30	3.35
45	Chlorogenic acid	C16 H18 O9	354.09534	17.071	5.02	0.21	0.22	12.10	6.32	12.99	6.71	13.79	9.04	11.12	1.40	30.40	52.80	69.58	5.80	5.19	6.34	5.63	4.94	5.22	4.65	4.53
47	Phenethyl Î²-primeveroside	C19 H28 O10	416.16868	17.176	116.12	27.20	18.94	23.34	35.29	5.96	14.69	26.09	13.39	1.97	13.06	127.20	262.55	151.17	20.08	17.78	19.71	17.11	16.02	18.64	16.23	15.69
49	Valproic acid beta-D-glucuronide	C14 H24 O8	320.14738	17.268	4.36	2.66	1.38	0.74	0.31	1.37	2.41	3.03	1.57	8.45	0.67	11.91	3.50	97.78	3.23	3.21	3.50	3.29	2.61	3.22	2.85	3.71
53	hydroxyethylidene)amino]-6-(hydroxymethyl)tetrahydro-2H-pyran-2-yl]oxy}-6-{2-[(2R,3S,4R,5R)-3,4-dihydroxy-5-(4-hydroxy-2-oxo-1(2H)-pyrimidinyl)tetrahydro-2-furanyl]-2-hydroxyethyl}-4,5-dihydroxytetrahydro-2H-pyran-3-yl]-11-methyl-2-dodecenimidic acid (non-	C36 H58 N4 O16	802.38405	17.629	5.04	0.10	6.41	5.31	5.63	3.66	5.68	11.45	5.84	3.57	9.40	37.47	23.58	17.69	5.16	4.27	4.59	4.46	3.50	3.74	3.53	3.61
62	Plantagoside	C21 H22 O12	466.11148	18.576	12.13	24.88	10.18	17.65	29.02	1.55	2.66	4.52	5.50	5.79	20.84	15.87	13.45	42.74	5.18	4.82	5.34	4.97	4.89	5.06	4.18	4.54
63	Procyanidin B2	C30 H26 O12	578.1427	18.93	213.49	560.70	0.01	69.62	28.93	191.21	566.46	0.01	1.62	1.89	347.92	701.35	0.38	926.72	173.95	138.24	127.35	109.64	123.64	82.73	140.12	111.84
68	Gentiopicrin	C16 H20 O9	356.11102	19.195	18.34	21.65	10.03	13.61	26.22	0.95	4.21	13.98	8.73	1.72	2.42	9.13	25.51	10.83	4.59	4.43	5.02	7.19	4.10	4.95	4.40	4.19
69	N-{[(2R,3S,4R,5S)-3,4-Dihydroxy-5-{2-[4-(2-methoxyphenyl)-1-piperazinyl]-2-oxoethyl}tetrahydro-2-furanyl]methyl}-4-(dimethylamino)benzamide	C27 H36 N4 O6	548.24723	19.375	72.16	29.42	63.29	78.57	106.43	27.59	37.31	45.41	30.16	67.17	94.22	115.93	80.05	174.37	27.28	26.45	28.20	27.35	23.06	29.57	23.16	24.21
73	Phenethyl-primeveroside	C19 H28 O10	416.16869	19.892	106.55	63.01	11.62	37.48	66.77	23.27	43.30	75.77	49.79	20.98	55.30	162.15	166.30	52.67	26.37	21.69	22.96	24.58	17.84	23.26	18.53	19.40
86	sinapoylglucose	C17 H22 O10	386.12188	21.274	8.87	1.37	7.74	4.37	6.49	0.57	1.32	29.95	10.46	5.18	5.38	22.61	46.52	17.97	2.82	2.84	4.33	3.05	2.63	3.35	2.98	2.64
87	Epicatechin	C15 H14 O6	290.07928	21.295	237.68	551.85	123.97	204.47	200.81	303.43	602.07	128.69	63.37	265.49	231.40	793.71	197.14	956.24	170.60	174.10	225.19	191.98	154.99	194.72	161.22	131.41
99	Indoleacetylaspartate	C14 H14 N2 O5	290.09057	22.348	17.26	0.10	29.48	73.00	16.48	62.87	22.95	229.79	18.96	56.57	236.95	32.35	187.65	66.45	20.47	16.59	21.60	17.31	19.41	25.89	19.14	18.13
102	3-Ethyl-4-hydroxy-4-methylpentyl 6-O-[(2S,3R,4R)-3,4-dihydroxy-4-	C19 H36 O11	440.22595	22.451	156.01	35.11	0.87	62.60	20.14	22.63	27.93	53.49	40.42	48.83	86.43	204.27	235.45	293.94	39.15	34.34	39.53	35.96	29.82	22.43	29.63	31.24
105	Epigallocatechin-3'-glucuronide	C21 H22 O13	482.10653	22.756	55.56	94.88	0.49	43.60	89.48	55.09	86.33	401.28	351.55	88.16	6.48	56.12	151.23	200.87	39.36	37.94	47.60	44.73	38.60	51.21	43.37	42.92
111	Abietin	C16 H22 O8	342.13173	23.175	7.76	2.05	0.01	1.57	1.86	21.01	0.80	18.57	11.32	10.92	0.44	34.53	17.19	12.53	6.20	5.46	6.24	6.01	4.69	5.87	4.73	4.18
112	3-Ethyl-4-hydroxy-4-methylpentyl 6-O-[(2S,3R,4R)-3,4-dihydroxy-4-	C19 H36 O11	440.2258	23.218	112.13	3.30	2.49	174.54	20.97	84.57	261.16	19.58	9.84	194.09	25.12	177.95	222.42	875.76	82.65	72.72	86.16	80.78	68.17	77.32	64.88	59.76
116	Furcatin	C20 H28 O10	428.16853	23.414	20.42	5.06	0.03	2.65	3.51	13.89	1.01	15.08	23.03	13.46	4.41	40.96	20.23	71.33	8.10	7.28	8.48	7.80	6.36	7.56	6.17	6.06
130	(2E)-3-Phenyl-2-propen-1-yl 6-O-beta-D-arabinofuranosyl-beta-D-	C20 H28 O10	428.1687	23.98	18.40	2.79	1.52	2.31	2.77	10.13	4.02	9.59	15.72	6.79	1.95	29.24	20.42	55.23	6.38	5.45	6.76	7.09	5.13	5.74	4.57	4.41
131	Astilbin	C21 H22 O11	450.11706	24.066	13.71	11.30	5.36	4.40	12.03	1.41	2.61	5.25	3.33	2.13	3.08	8.64	19.19	10.86	3.13	2.75	3.34	3.04	2.57	3.06	2.39	2.45
136	Lusitanicoside	C21 H30 O10	442.18454	24.417	10.31	2.17	2.65	2.62	7.58	2.14	3.06	8.49	18.80	7.76	1.88	48.23	10.41	47.07	5.56	4.99	5.61	5.56	4.39	5.08	3.82	3.75
145	7-Hydroxy-2-(4-hydroxyphenyl)-4-oxo-3,4-dihydro-2H-chromen-5-yl β-D-	C21 H22 O10	434.12199	25.057	13.85	12.23	8.00	9.77	19.88	3.91	6.43	20.00	15.79	8.66	10.10	21.46	20.72	22.42	5.79	6.35	5.06	4.04	4.82	6.62	5.35	4.65
147	2-(3,4-Dihydroxyphenyl)-5,7-dihydroxy-4-oxo-4H-chromen-3-yl 6-O-β-D-	C26 H28 O16	596.1387	25.161	7.63	0.88	1.87	3.74	15.43	7.04	1.86	56.95	19.22	0.29	4.41	18.37	14.15	11.96	3.93	3.71	3.46	4.04	2.90	3.61	2.76	2.67
148	beta-Syringin	C17 H24 O9	372.14252	25.193	7.32	5.30	5.55	4.60	8.92	2.65	5.53	5.89	10.11	4.56	2.49	10.18	10.22	19.25	3.07	2.72	3.25	3.24	2.60	3.29	2.45	2.56
152	Similar to Catechin	C15 H14 O6	290.0793	25.477	33.42	22.59	6.90	6.36	12.87	3.40	10.82	9.83	8.92	4.03	6.22	55.86	41.32	28.92	9.09	7.52	8.91	8.08	6.77	8.03	6.69	6.18
156	Quercetin-3β-D-glucoside	C21 H20 O12	464.09606	25.59	156.04	105.12	128.09	177.42	181.98	85.07	176.91	352.42	243.46	155.73	166.32	352.68	400.70	222.41	90.89	57.76	101.03	80.12	87.12	67.91	79.69	90.63
159	Rutin	C27 H30 O16	610.15404	25.674	50.61	26.24	47.59	82.06	104.63	30.26	9.55	85.09	37.14	78.07	67.72	135.84	115.69	68.13	29.76	27.67	30.05	31.04	26.25	29.82	22.67	21.24
161	3,5-Dihydroxy-2-[3-(4-hydroxyphenyl)propanoyl]phenyl 2-O-beta-D-	C26 H32 O14	568.17955	25.844	381.03	97.13	44.36	147.93	70.70	190.12	345.52	261.83	119.46	187.56	209.22	751.50	686.31	889.49	154.50	121.79	140.08	126.63	110.14	146.55	108.14	107.21
166	Astragalin	C21 H20 O11	448.10097	26.116	5.31	2.38	5.91	18.57	39.92	2.94	2.50	7.31	4.44	11.13	4.43	11.93	11.23	15.20	6.16	5.68	6.03	6.85	4.72	5.59	3.78	4.51
170	Phloretin	C15 H14 O5	274.08427	26.224	50.24	16.27	9.06	11.70	11.45	4.60	10.88	12.51	7.63	4.51	9.75	59.68	51.88	63.78	7.52	9.75	10.45	11.07	7.89	8.83	8.35	6.79
183	Juglalin	C20 H18 O10	418.0905	26.725	14.09	5.45	11.15	16.02	51.05	8.94	7.63	11.03	7.08	9.62	26.07	39.61	24.78	29.06	8.46	7.25	8.63	7.74	6.37	7.37	5.52	5.75
190	Afzelin	C21 H20 O10	432.10623	27.165	43.77	9.04	10.89	14.72	59.18	8.77	32.58	10.30	5.96	15.70	20.75	70.87	86.56	42.36	9.31	7.39	9.03	8.28	6.79	8.16	6.04	5.53
192	Quercetin	C15 H10 O7	302.04295	27.871	52.57	7.78	4.10	5.49	8.21	9.62	8.74	7.23	8.62	8.40	7.25	61.51	103.72	246.54	19.13	16.32	15.58	17.40	13.45	16.32	14.08	13.39
193	Phloionolic acid	C18 H36 O5	332.25612	28.568	9.35	2.87	0.78	0.78	0.83	0.48	2.80	33.86	17.52	0.61	1.20	3.01	51.63	35.13	3.26	3.05	3.50	3.35	2.86	3.78	3.07	3.14
195	Luteolin	C15 H10 O6	286.04804	28.843	16.20	12.80	14.80	13.49	14.66	14.04	16.23	10.30	12.11	9.37	10.49	13.92	9.26	18.11	12.13	10.99	14.43	12.28	11.73	15.05	13.46	12.70
196	2,3-Dihydroxyolean-12-ene-23,28-dioic acid	C30 H46 O6	502.32997	29.671	19.23	3.46	1.31	2.50	15.18	4.24	1.99	8.43	3.78	1.13	0.05	136.76	190.95	44.29	9.18	7.14	8.21	6.57	6.21	7.21	6.51	7.42
199	Fludioxonil	C12 H6 F2 N2 O2	248.0403	30.396	64.02	3.81	0.02	0.01	0.04	0.09	0.01	257.81	127.18	2.58	0.04	71.43	192.09	4.65	16.62	15.03	19.49	18.36	16.79	22.51	20.10	20.46
201	Dodecyl sulfate	C12 H26 O4 S	266.15531	30.879	15.13	13.16	8.05	8.85	8.18	3.78	8.01	13.88	8.73	5.04	10.50	19.83	12.86	15.82	4.15	3.89	4.31	4.24	3.27	4.59	3.60	3.56
205	4-Undecylbenzenesulfonic acid	C17 H28 O3 S	312.17612	31.802	9.44	6.61	8.28	7.12	7.25	3.45	6.93	11.38	7.75	5.52	6.82	17.71	11.34	16.29	3.55	3.10	3.29	3.67	2.65	3.71	2.61	2.70
209	3-O-Isobutyryl-beta-D-fructofuranosyl 3-O-dodecanoyl-4-O-(3-	C33 H58 O14	678.3836	32.069	8.64	0.43	0.54	0.32	1.03	0.06	0.32	0.45	0.29	0.04	0.42	29.48	43.23	29.74	4.03	2.83	2.84	2.80	2.65	3.22	2.84	3.31
220	Lexibulin	C24 H30 N6 O2	434.24377	32.588	50.20	1.55	1.21	1.04	1.74	0.73	2.34	4.21	1.91	0.50	2.86	68.56	185.01	60.84	8.93	7.13	7.21	7.23	7.51	10.64	7.31	8.75
221	1-stearyl estercitric acid	C24 H44 O7	444.30908	32.593	3.54	0.12	3.48	19.24	22.55	0.01	4.67	0.02	0.02	0.11	0.04	9.09	15.01	2.30	2.72	2.50	2.62	2.50	2.43	2.78	2.40	2.54
226	18-β-Glycyrrhetinic acid	C30 H46 O4	470.34054	32.871	17.33	8.67	19.47	22.28	44.97	0.52	21.63	15.72	13.14	1.48	0.51	95.68	107.95	47.34	16.37	8.62	14.33	14.78	11.72	13.18	14.88	15.55
231	2-Hydroxy-3-{[(2E)-3-(4-hydroxyphenyl)-2-propenoyl]oxy}urs-12-en-28-oic	C39 H54 O6	618.39254	33.307	31.77	24.17	13.11	9.07	9.70	0.32	11.48	14.05	15.82	0.28	3.18	72.01	93.91	57.23	11.81	11.59	13.43	12.52	8.76	13.39	11.23	14.37
236	2-Hydroxy-3-{[(2E)-3-(4-hydroxyphenyl)-2-propenoyl]oxy}urs-12-en-28-oic	C39 H54 O6	618.39257	33.758	4.62	1.16	7.20	1.66	2.16	0.17	2.27	1.70	0.53	0.13	0.34	31.93	17.99	29.12	5.51	3.87	3.58	3.98	3.64	3.50	4.04	4.36
